# Occurrence and Risk Factors of Adverse Drug Reactions in Patients Receiving Bivalirudin as Anticoagulant During Percutaneous Coronary Intervention: A Prospective, Multi-Center, Intensive Monitoring Study

**DOI:** 10.3389/fcvm.2021.781632

**Published:** 2022-04-29

**Authors:** Ping Li, Hongyan Zhang, Caidong Luo, Zheng Ji, Zeqi Zheng, Zhenyong Li, Fan Wu, Jinlong Li, Lang Hong

**Affiliations:** ^1^Department of Cardiology, The First People's Hospital of Yulin, Yulin, China; ^2^Department of Cardiology, Affiliated Hospital of Qilu Medical University (The People's Hospital of Xin Tai City), Xintai, China; ^3^Department of Cardiology, Mianyang Central Hospital, Mianyang, China; ^4^First Department of Cardiology, Tangshan Workers' Hospital, Tangshan, China; ^5^Department of Cardiology, The First Affiliated Hospital of Nanchang University, Nanchang, China; ^6^Department of Cardiology, Xuzhou Central Hospital, Xuzhou, China; ^7^Second Department of Cardiology, Xuchang Central Hospital, Xuchang, China; ^8^Department of Cardiology, The Affiliated Taian City Central Hospital of Qingdao University, Tai'an, China; ^9^Jiangxi Provincial People's Hospital, The First Affiliated Hospital of Nanchang Medical College, Nanchang, China

**Keywords:** bivalirudin, percutaneous coronary intervention, adverse events, adverse drug reactions, bleeding and thrombocytopenia

## Abstract

**Background:**

Bivalirudin is a common anticoagulant during percutaneous coronary intervention (PCI); however, since its application in China, it still lacks comprehensive evaluation of adverse events (AEs) or adverse drug reactions (ADRs) under the real-clinical setting conditions with a large-sample-size population. Therefore, this prospective, multi-center, intensive monitoring study aimed to comprehensively investigate the occurrence and risk factors of AEs and ADRs during PCI with bivalirudin as an anticoagulant.

**Methods:**

A total of 3,049 patients who underwent PCI with bivalirudin as anticoagulant from 27 Chinese medical centers were enrolled. Safety data (AEs/ADRs) were collected from hospital admission to 72 h after bivalirudin administration; then, patients were followed up at the 30th day with the safety data collected as well.

**Results:**

A total of 414 (13.58%) patients occurred AEs, among which 31 (1.02%) cases suffered from severe AEs and 8 (0.26%) cases died due to AEs. Importantly, 118 (3.87%) patients occurred bivalirudin related ADRs, among which 7 (0.23%) cases suffered from severe ADRs while no case (0%) died due to ADRs. Of note, 7 (0.23%) patients showed new ADRs, 34 (1.12%) patients experienced bleeding, and 79 (2.59%) patients had thrombocytopenia. Furthermore, age, renal function impairment, CRUSADE high risk stratification independently correlated with total ADRs risk; CRUSADE high risk stratification, emergency operation, full dose bivalirudin independently associated with bleeding risk; age, renal function impairment independently related to thrombocytopenia risk.

**Conclusion:**

Bivalirudin is well-tolerated as an anticoagulant for PCI procedure; meanwhile, older age, renal function impairment, and CRUSADE high risk stratification serve as independent risk factors of bivalirudin related ADRs.

## Introduction

Cardiovascular disease, as the most common cause of mobility and mortality, is a critical issue affecting global human health (1). Pleasingly, along the introduction and advancement of percutaneous coronary intervention (PCI), the outcomes and long-term prognosis of patients with cardiovascular disease have been improved a lot, while its concomitant complications still worry the physicians and patients (2, 3). Among PCI-related complications, thrombotic complication such as stent thrombosis, is one of the prior concerns to be handled; then for years, unfractionated heparin (UFH) (with or without glycoprotein IIb/IIIa inhibitors) was considered as the anticoagulant choice during PCI, while it exhibits several limits: unpredictability of biological efficiency, platelets activation, heparin-induced thrombopenia, and bleeding (4–7). Therefore, exploration of better adjunctive anticoagulant during PCI is of great importance.

Bivalirudin, a synthetic analog of natural anticoagulant hirudin which consists of 20 amino acid peptides, is an explicit and irreversible thrombin inhibitor with concentration-dependent competitive and non-competitive thrombin repressing effect (8, 9). Unlike the UFH, bivalirudin not only realizes expectable anticoagulant outcome with linear pharmacokinetic and pharmacodynamic properties, it also achieves quicker onset and has a shortened action period; but also reduces the influence of platelet function and bleeding, which makes it a potentially alternative option for anticoagulant during PCI (8–12). Clinically, several randomized trials have demonstrated the non-inferiority of bivalirudin vs. UFH plus glycoprotein IIb/IIIa inhibitors regrading ischemic events, but the superiority of lower bleeding risk in PCI procedures as anticoagulant (6, 12–14). However, since its application in China, it still lacks comprehensive evaluation of adverse events (AEs) or adverse drug reactions (ADRs) under the real-clinical setting conditions with a larger-sample-size population, especially for the exploration of severe, rare, or new ones; furthermore, identification of their risk factors is deficient as well.

Therefore, the current prospective, multi-center, intensive monitoring study enrolled 3,049 patients who underwent PCI with bivalirudin as anticoagulant from 27 Chinese medical centers, then aimed to comprehensively investigate the occurrence and risk factors of AEs and ADRs, especially for bleeding and thrombocytopenia.

## Methods

### Study Design

This was a prospective, multi-center, intensive monitoring study. The case enrollment and information collection of this study were carried out in 27 Chinese medical centers (shown in Supplementary Table 1). In the whole process of the study, the medication use and treatment of patients were determined by attending physicians according to the actual clinical situation, which were not interfered by the study. This study was approved by the Ethics Committee of each participant center with those of Beijing Anzhen Hospital Affiliated to Capital Medical University (approval number: KS2019012) and Peking University People's Hospital (approval number: 2018PHA092-001) as the primary Ethics Committees.

### Patient Enrollment

This study was conducted between July 2018 and June 2019. To observe a 0.1% incidence of ADRs (that was, ARDs occurred in at least one or more cases), the required sample size was 3,000, with a power of 95%. A total of 3,050 patients were recruited, among which, one patient was excluded subsequently due to that the patient only received an angiography without PCI. Finally, 3,049 patients who underwent PCI with bivalirudin used as anticoagulant in the 27 medical centers were enrolled and analyzed in the current study. The inclusion criteria were: (1) underwent PCI or percutaneous coronary angioplasty (PTCA); (2) using bivalirudin as anticoagulant; (3) age over 18 years; (4) understand the study content and voluntarily participated in the study. Patients without use of bivalirudin were excluded from the study.

### Clinical Data Collection

Clinical data were collected after hospital admission. The mainly collected data covered the following: (1) basic information: age, gender, height, and weight; (2) medical history: allergy, cardiac surgery, critical respiratory disease, diabetes or thrombolysis, and tumor; (3) renal function impairment and severity; (4) clinical features: clinical presentation, CRUSADE score (Can Rapid Risk Stratification of Unstable Angina Patients Suppress Adverse Outcomes with Early Implementation of the ACC/AHA Guidelines), and CRUSADE risk stratification; (5) PCI-related information: timing limitation of operation (emergency or elective), types of coronary interventional therapy, arterial access, operative time, and time of bivalirudin infusion; (6) treatment: full-dose use of bivalirudin, and glycoprotein IIb/IIIa inhibitor use; (7) Thrombolysis in Myocardial Infarction (TIMI) grade: pre-procedure TIMI grade, and post-procedure TIMI grade.

### Safety Data Collection

Safety data were collected from hospital admission to 72 h after completion of bivalirudin administration. In addition, patients were followed up at the 30th day, and the data were also collected at that time. The primary safety data included any AEs, which were required to be documented in detail during the period of intensive monitoring. Classification of AEs was coded using the Systematic Organ Classification (SOC) and Preferred Term (PT) of the International Conference on the Coordination of International Drug Registration (ICH) International Dictionary of Medical Terms (MedDRA) 23.0. The intensive monitoring ADRs were bleeding and thrombocytopenia, among which, the bleeding was graded in terms of Bleeding Academic Research Consortium (BARC) consensus classification criteria (15). All the summaries of AEs were based on cases. If a case suffered from the same AE repeatedly, the severity of the AE was calculated according to the most serious one.

### Definitions

AEs were defined as any unfavorable and unintended sign (including an abnormal laboratory finding), symptom, or disease temporally associated with the use of a medical treatment that may or may not be considered related to the medical treatment. Severe adverse events (SAEs) were defined as one of the following events: (1) leading to death; (2) life-threatening consequences; (3) leading to carcinogenesis, teratogenesis, and birth defects; (4) leading to significant or permanent human disability or organ function damage; (5) resulting in hospitalization or prolonged length of stay; (6) leading to other important medical events, if not treated, the above-listed conditions may occur. ADRs were defined as the harmful reaction of qualified drugs which was irrelevant to the purpose of medication under normal usage and dosage. New ADRs were defined as the adverse reactions not specified in the drug instructions. Moreover, if the nature, extent, consequence, or frequency of adverse reactions were inconsistent with the drug instructions but more serious than those described in the instructions, they were also regarded as new ADRs.

### Statistical Analysis

**Figure 1:**
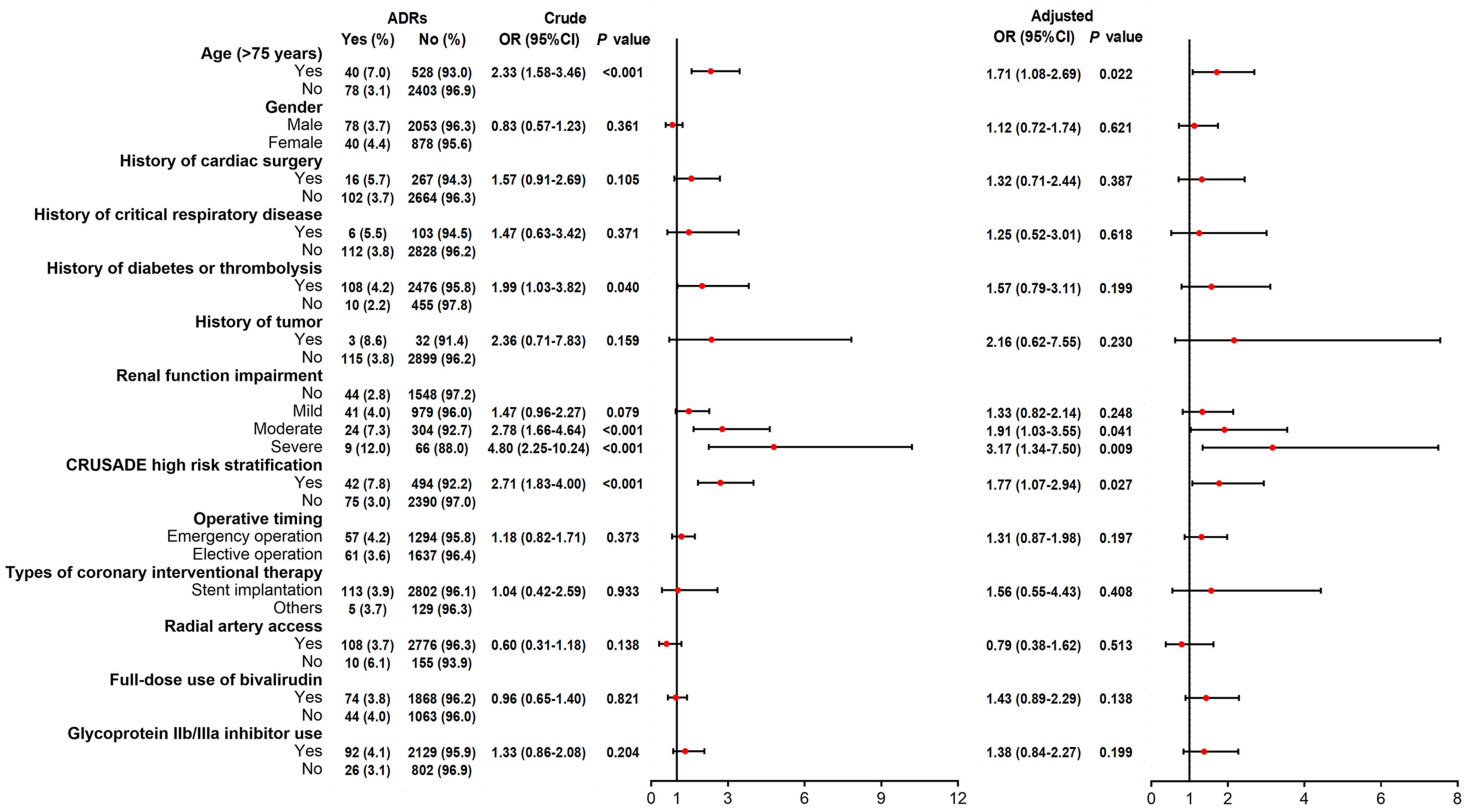
Logistic regression analyses for factors related to ADRs risk.

**Figure 2:**
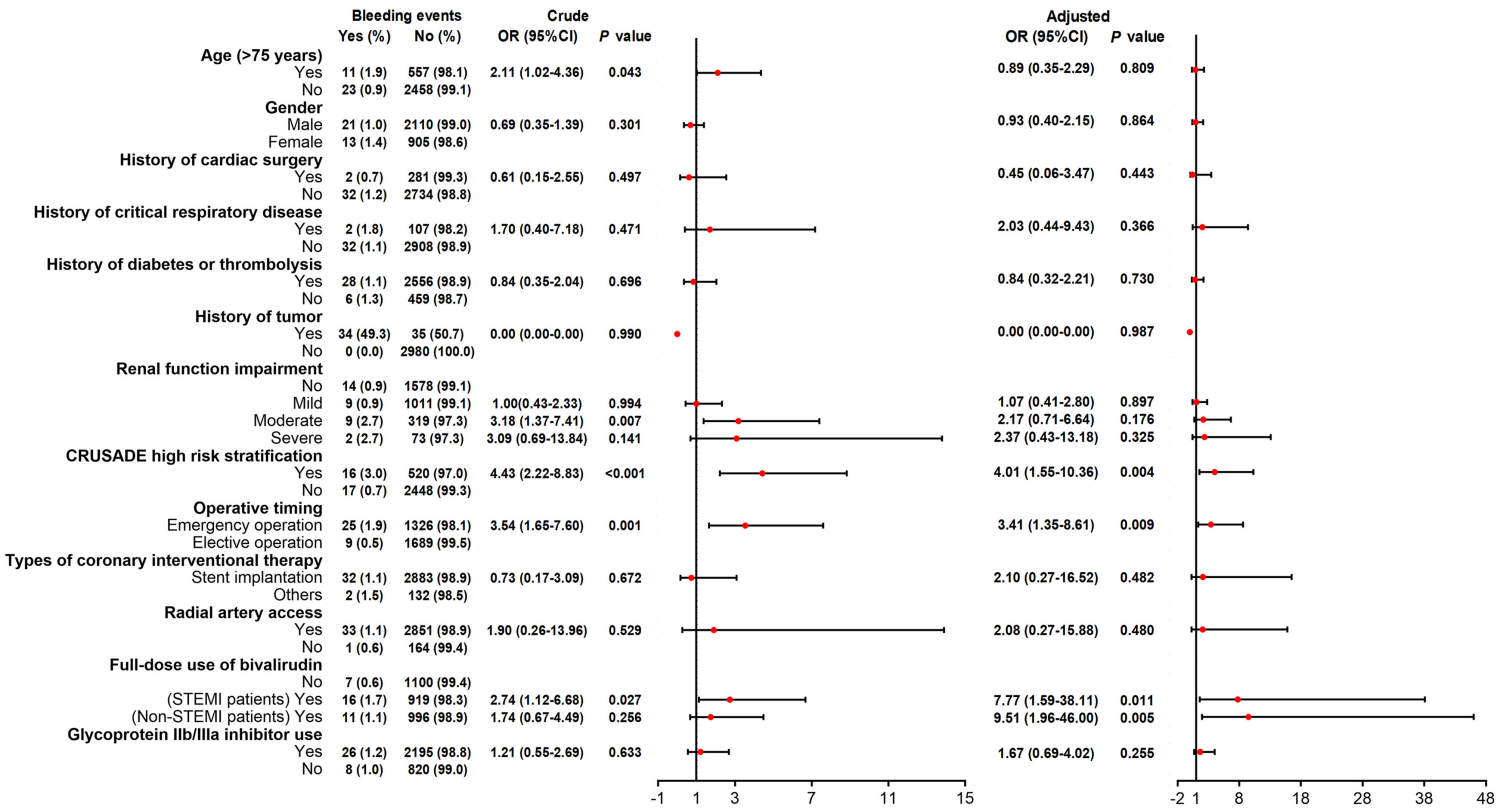
Logistic regression analyses for factors predicting bivalirudin related bleeding events risk.

**Figure 3:**
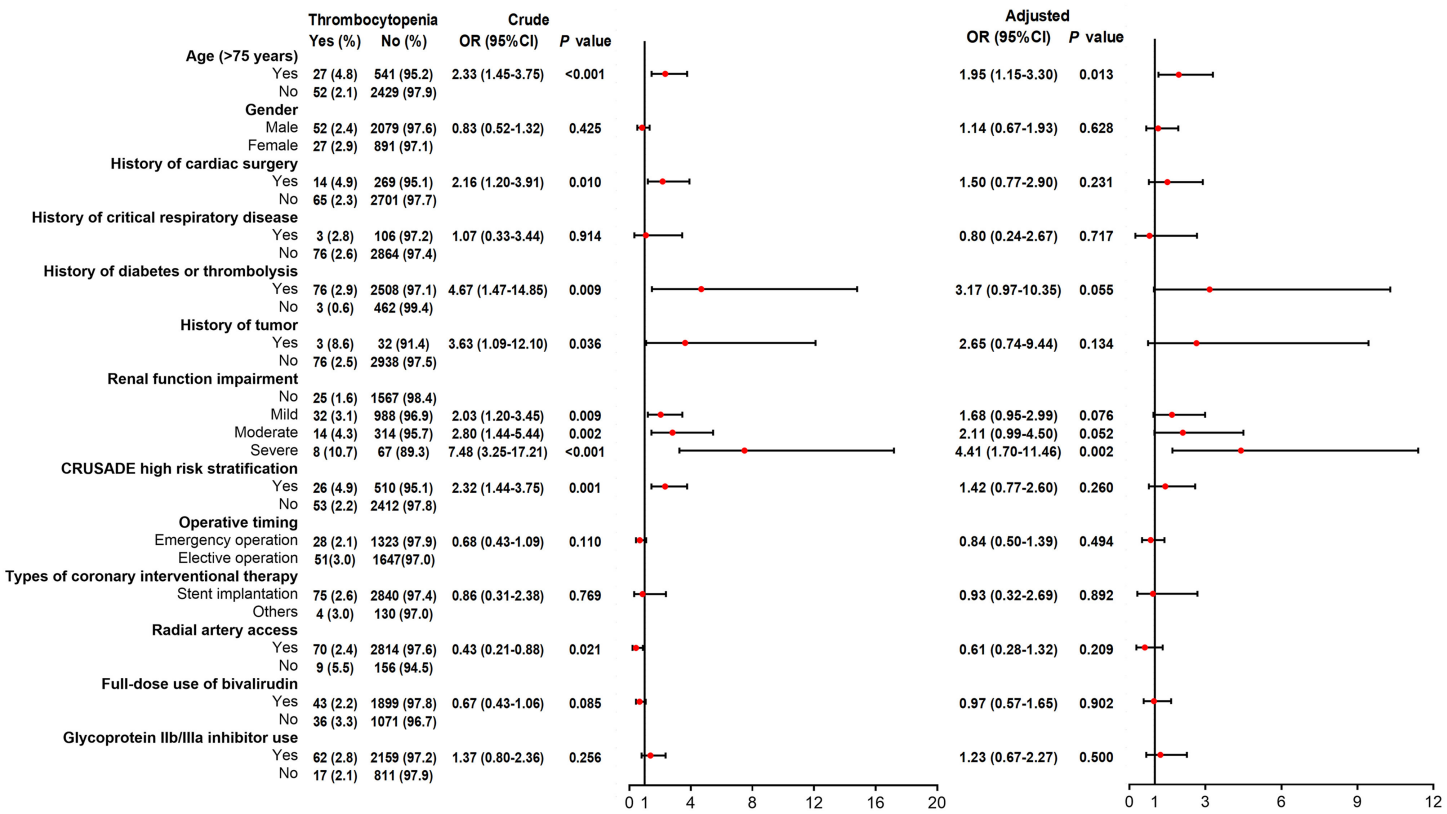
Logistic regression analyses for factors of bivalirudin related thrombocytopenia risk.

SAS 9.4 (SAS Institute, Inc., Cary, NC) was applied to complete data analysis. Continuous variables were expressed as mean ± standard deviation (SD), and categorical variables were described as times, numbers, and percentages, as well as 95% confidence intervals (CI). Summary of AEs and ADRs was presented as number of times, incidence rate, and 95%CI. Detailed AEs, ADRs, and new ADRs in SOC were described as the number of times and incidence rate. Incidence of ADRs (Figure 1), bleeding events (Figure 2), and thrombocytopenia (Figure 3) in different populations was checked by Chi-square test or Fisher's exact test (when the expected count was less than 5). The crude odds ratio (OR) in Figures 1–3 was estimated by univariate logistic regression model analysis, and the corresponding adjusted OR was estimated by multivariate logistic regression model analysis with all variables included in, which were shown in a forest plot. A *P* < 0.05 was regarded as significant significance.

## Results

### Characteristics of Patients and PCI Procedure

The enrolled patients exhibited a mean age of 64.78 ± 11.39 years with 2,131 (69.89%) males and 918 (30.11%) females. There were 1,244 (40.81%), 414 (13.58%), 1,034 (33.91%), and 352 (11.54%) patients who showed clinical presentation of ST-segment elevation myocardial infarction (STEMI), non-ST-segment elevation myocardial infarction (NSTMI), unstable angina (UA), and spontaneous coronary artery dissection (SCAD), respectively; while 5 (0.16%) showed unknown clinical presentation. Furthermore, CRUSADE risk stratification identified 722 (23.68%), 1,014 (33.26%), 729 (23.91%), 342 (11.22%), 194 (6.36%), and 48 (1.57%) as very low risk, low risk, moderate risk, high risk, very high risk, and unknown risk, respectively. The detailed characteristics of patients are shown in Table 1.

**Table 1:** Clinical characteristics of participants.

**Parameters**	**Patients (*N* = 3,049)**
Age (years), mean ± SD	64.78 ± 11.39
Gender, No. (%)	
Male	2,131 (69.89)
Female	918 (30.11)
Height (cm)	
Assessable, No. (%)	2,852 (93.54)
Mean ± SD	165.25 ± 8.28
Weight (kg)	
Assessable, No. (%)	2,995 (98.22)
Mean ± SD	66.35 ± 11.56
History of allergy, No. (%)	274 (8.99)
History of cardiac surgery, No. (%)	283 (9.28)
History of critical respiratory disease, No. (%)	109 (3.57)
History of diabetes or thrombolysis, No. (%)	2,584 (84.75)
History of tumor, No. (%)	35 (1.15)
Renal function impairment, No. (%)	
No	1,592 (52.21)
Mild	1,020 (33.45)
Moderate	328 (10.76)
Severe	75 (2.46)
Unknown	34 (1.12)
Clinical presentation, No. (%)	
STEMI	1,244 (40.81)
NSTMI	414 (13.58)
UA	1,034 (33.91)
SCAD	352 (11.54)
Unknown	5 (0.16)
CRUSADE score	
Assessable, No. (%)	3,001 (98.43)
Mean ± SD	29.42 ± 13.09
CRUSADE risk stratification, No. (%)	
Very low risk	722 (23.68)
Low risk	1,014 (33.26)
Moderate risk	729 (23.91)
High risk	342 (11.22)
Very high risk	194 (6.36)
Unknown	48 (1.57)

*SD, standard deviation; STEMI, ST-segment elevation myocardial infarction; NSTMI, non-ST-segment elevation myocardial infarction; UA, unstable angina; SCAD, spontaneous coronary artery dissection; CRUSADE, Can Rapid Risk Stratification of Unstable Angina Patients Suppress Adverse Outcomes with Early Implementation of the ACC/AHA Guidelines*.

Regarding the characteristics of PCI, 1,351 (44.31%) performed emergency operation while 1,698 (55.69%) performed elective operation. Operative duration and duration of bivalirudin infusion were 69.60 ± 48.00 min and 91.15 ± 77.40 min, respectively. Besides, 1,942 (63.69%) and 2,221 (72.84%) had full-dose use of bivalirudin and glycoprotein IIb/IIIa inhibitor use, respectively. The detailed characteristics of the PCI procedure are shown in Supplementary Table 2. Furthermore, so as to clearly present the clinical administration of bivalirudin in the current study, the detailed information was also listed (Supplementary Table 3).

### General Information of AEs and ADRs

A total of 414 (13.58%) patients occurred AEs, among which 31 (1.02%) cases suffered from SAEs and 8 (0.26%) cases died due to AEs (Table 2). Importantly, regarding bivalirudin, a total of 118 (3.87%) patients occurred ADRs, among which 7 (0.23%) cases suffered from SADRs while no case (0%) died due to ADRs (Table 2). Furthermore, 7 (0.23%) patients showed new ADRs of bivalirudin, 34 (1.12%) patients experienced bleeding, and 79 (2.59%) patients had thrombocytopenia.

**Table 2:** Summary of AEs and ADRs.

**Items**	**Number of times**	**Incidence, no. (%)**	**95% CI of incidence**
**Adverse events (AEs)**			
Total AEs	829	414 (13.58)	12.38–14.84
SAEs	38	31 (1.02)	0.69–1.44
Death due to AEs	9	8 (0.26)	0.11–0.52
**Adverse drug reactions (ADRs)**			
Total ADRs	130	118 (3.87)	3.21–4.62
SADRs	7	7 (0.23)	0.09–0.47
Death due to ADRs	0	0 (0.00)	–
New ADRs	8	7 (0.23)	0.09–0.47
Bleeding	37	34 (1.12)	0.77–1.55
BARC type 0	1	1 (0.03)	0.00–0.18
BARC type 1	29	27 (0.89)	0.58–1.29
BARC type 2	2	2 (0.07)	0.01–0.24
BARC type 3a	5	4 (0.13)	0.04–0.34
Thrombocytopenia	79	79 (2.59)	2.06–3.22

*AEs, adverse events; SAEs, severe adverse events; ADRs, adverse drug reactions; SADRs, severe adverse drug reactions; BARC, Bleeding Academic Research Consortium; CI, confidence interval*.

### Detailed AEs and ADRs Categorized by System Organ Class (SOC)

The most common AEs were gastrointestinal disorders (3.58%), general disorders and administration site conditions (3.31%), respiratory, thoracic, and mediastinal disorders (3.18%), then blood and lymphatic system disorders (2.66%) (Supplementary Table 4). Meanwhile, the most prevalent ADRs of bivalirudin were blood and lymphatic system disorders (2.62%), gastrointestinal disorders (0.69%), investigations (0.20%), then respiratory, thoracic, and mediastinal disorders (0.16%) (Table 3). Specifically, the detailed information of total bleeding events and bleeding events related to bivalirudin are presented in Supplementary Table 5. In addition, 6 patients experienced one new ADR and 1 patient faced two new ADRs, resulting in 7 (0.23%) patients suffering from new ADRs (Supplementary Table 6). The detailed information of AEs, ADRs, and new ADRs could be referred to Table 3 and Supplementary Tables 4, 6, respectively.

**Table 3:** Detailed ADRs in System Organ Class (SOC).

**Items**	**Total**		**SADRs**		**Death due to ADRs**	
	**Number of times**	**Incidence, no. (%)**	**Number of times**	**Incidence, no. (%)**	**Number of times**	**Incidence, no. (%)**
Total	130	118 (3.87)	7	7 (0.23)	0	0 (0.00)
Blood and lymphatic system disorders	80	80 (2.62)	0	0 (0.00)	0	0 (0.00)
Gastrointestinal disorders	24	21 (0.69)	5	5 (0.16)	0	0 (0.00)
Investigations	6	6 (0.20)	0	0 (0.00)	0	0 (0.00)
Respiratory, thoracic, and mediastinal disorders	6	5 (0.16)	0	0 (0.00)	0	0 (0.00)
Renal and urinary disorders	4	4 (0.13)	0	0 (0.00)	0	0 (0.00)
Skin and subcutaneous tissue disorders	3	3 (0.10)	0	0 (0.00)	0	0 (0.00)
General disorders and administration site conditions	2	2 (0.07)	2	2 (0.07)	0	0 (0.00)
Injury, poisoning, and procedural complications	2	1 (0.03)	0	0 (0.00)	0	0 (0.00)
Musculoskeletal and connective tissue disorders	1	1 (0.03)	0	0 (0.00)	0	0 (0.00)
Cardiac disorders	1	1 (0.03)	0	0 (0.00)	0	0 (0.00)
Nervous system disorders	1	1 (0.03)	0	0 (0.00)	0	0 (0.00)

*ADRs, adverse drug reactions; SADRs, severe adverse drug reactions*.

### Risk Factors Related to ADRs

Univariate logistic regression model identified that age (>75 years), history of diabetes or thrombolysis, renal function impairment (moderate or severe), and CRUSADE high risk stratification correlated with increased ADRs occurrence (Figure 1). Then after adjustment by multivariate logistic regression model, age (>75 years) (OR 1.71, 95%CI 1.08–2.69; *P* = 0.022), renal function impairment (moderate OR 1.91, 95%CI 1.03–3.55; *P* = 0.041) (severe OR 3.17, 95%CI 1.34–7.50; *P* = 0.009), and CRUSADE high risk stratification (OR 1.77, 95%CI 1.07–2.94; *P* = 0.027) were discovered to dependently relate to ADRs occurrence (Figure 1).

### Risk Factors of Bivalirudin Related Bleeding Events

Univariate logistic regression model disclosed that age (>75 years), moderate renal function impairment, CRUSADE high risk stratification, operative timing (emergency vs. elective), and full dose use of bivalirudin in STEMI patients correlated with increased bivalirudin related bleeding events occurrence (Figure 2). After adjustment by multivariate logistic regression model, CRUSADE high risk stratification (OR 4.01, 95%CI 1.55–10.36; *P* = 0.004), operative timing (emergency vs. elective) (OR 3.41, 95%CI 1.35–8.61; *P* = 0.009), full dose use of bivalirudin (STEMI patients OR 7.77, 95%CI 1.59–38.11; *P* = 0.011) (Non-STEMI patients OR 9.51, 95%CI 1.96–46.00; *P* = 0.005) independently correlated with increased bivalirudin related bleeding events occurrence (Figure 2).

### Risk Factors of Bivalirudin Related Thrombocytopenia

Univariate logistic regression model exhibited that age (>75 years), history of cardiac surgery, history of diabetes or thrombolysis, history of tumor, renal function impairment (mild, moderate, or severe vs. no), CRUSADE high risk stratification, and radial artery access correlated with thrombocytopenia risk (Figure 3). Post adjustment via multivariate logistic regression model, only age (>75 years) (OR 1.95, 95%CI 1.15–3.30; *P* = 0.013) and renal function impairment (severe OR 4.41, 95%CI 1.70–11.46; *P* = 0.002) were independently associated with elevated thrombocytopenia risk (Figure 3).

## Discussions

Accumulating reports demonstrate that anticoagulant use of bivalirudin during PCI is of very good tolerance, while some minor AEs or ADRs still need attention, such as bleeding (especially major bleeding), cardiac/cardiovascular events (such as heart failure, cardiogenic shock, recurrent myocardial infarction, etc.), cerebrovascular events (such as stroke, etc.), stent thrombosis, and so on (16–21). It was reported that bleeding events occur in 5.0–11.3% patients, among which major bleeding events affect 0.9–7.8% patients who are undergoing PCI with bivalirudin as anticoagulant (16, 19, 22). Furthermore, safety related major adverse cardiac events (MACEs) appear in 7.6–13.4% patients (17, 19); safety related stroke events happen in 0.2–1.7% patients who perform bivalirudin-involving PCI procedure (16, 17, 22). In addition, 0.7–2.4% bivalirudin-involving PCI treated patients suffer from stent thrombosis events (19, 22, 23). However, the general, detailed evaluation of AEs regarding bivalirudin application as anticoagulant in PCI is still limited; not to mention that it also lacks comprehensive assessment of AEs under the real-clinical setting conditions with a large sample size population in China. In the current prospective, multi-center, large sample size, intensive monitoring study, we observed that 13.58% patients underwent PCI with anticoagulant bivalirudin occurred AEs, among which 1.02% cases suffered from SAEs and 0.26% cases died due to AEs. Since no previous report discusses the comprehensive AEs regarding PCI with anticoagulant bivalirudin, the percentage could not be compared or referred to. Then after categorization by SOC, the most common AEs were gastrointestinal disorders (3.58%), general disorders and administration site conditions (3.31%), respiratory, thoracic, and mediastinal disorders (3.18%), and then blood and lymphatic system disorders (2.66%). These data inspired us to pay attention to and even prevent these AEs in advance. In terms of bleeding, 4.13% studied patients experienced bleeding events, which was numerically lower compared to that in previous studies (16, 19, 22), which might result from: (1) the improved knowledge of the studied drug and advanced procedure technology and skills; and (2) the different study design, enrolled patients, observational period, and so on.

In order to provide the precise and strict information about the adverse events/adverse reactions related to bivalirudin application as anticoagulant during PCI for further drug choice guiding, we evaluated ADRs of bivalirudin during PCI, which were defined as the harmful reaction of qualified drugs that was irrelevant to the purpose of medication under normal usage and dosage. It was discovered that 3.87% patients experienced bivalirudin related ADRs, among which 0.23% cases suffered from SADRs, while no case died due to ADRs. However, there has been no previous study analyzing the bivalirudin related ADRs during PCI. This current study is the first to provide the overall information about it (16–23). Generally speaking, we observed that bivalirudin was well-tolerated as an anticoagulant for PCI. Then to identify potential risk factors of ADRs induced by bivalirudin during PCI, logistic regression analysis was performed which discovered that older age, renal function impairment, and CRUSADE high risk stratification independently correlated with ADRs occurrence. The possible explanations were as follows: (1) Patients with older age might have more complications and worse metabolism ability for drugs, leading to increased ADRs (24, 25); (2) renal function impairment affected the drug metabolism directly, which resulted in ADRs (26); (3) CRUSADE high risk stratification reflected higher possibility of bleeding, which was one of the most common ADRs of bivalirudin. In the current study, one patient died of acute heart failure, two patients died of acute myocardial infraction, two patients died of tachycardia, one patient died of hypotension, one patient died of hypoxemia, and two patients died of systematic syndromes. After assessment, it is considered that none of these patients died of bivalirudin-related adverse reactions.

Furthermore, 0.23% patients showed new ADRs of bivalirudin, 1.12% patients experienced bivalirudin related bleeding, and 2.59% patients had bivalirudin related thrombocytopenia during PCI. These informed us that the occurrence of bleeding and thrombocytopenia, directly induced by bivalirudin, were less than the previous opinions (16, 19, 22). Moreover, apart from CRUSADE high risk stratification, operative timing (emergency vs. elective), and full dose use of bivalirudin also independently correlated with increased bivalirudin related bleeding events risk during PCI. These might result from: (1) an emergency operation reflected the acute and severe disease condition or short preparation time for complication prevention therefore led to increased bleeding risk; (2) full dose vs. reduced dose use of bivalirudin suggested the increased anticoagulant effect, generating increased bleeding possibility. When looking at thrombocytopenia, older age and renal function impairment were independently associated with its increased risk, with the possible explanations the same as their contributions to total ADRs risk (24–26). In addition, it is of note that a previous HEAT-PPCI study observes that bivalirudin does not decrease bleeding risk while increases acute stent thrombosis risk compared to UFH in STEMI patients (27). However, it brings about great dispute resulting from the following issues: (1) HEAT-PPCI is a single-center study; (2) the assessment of AEs by a third party is not carried out; and (3) activated clotting time (ACT) during operation is quite accidentally lower compared to previous studies. The findings of this study confirmed that bivalirudin as an anti-coagulant during PCI was a safe regimen. Meanwhile, the recognition of risk factors related to ADRs, bleeding, and thrombocytopenia might help to improve the administration of bivalirudin as anti-coagulant during PCI.

Despite these interesting findings, the current study still faces some limitations. First, the sample size of this study was calculated and set to 3,049 based on the purpose to observe the ADR with at least 0.1% incidence, while some SADRs or even deadly ADRs might be below 0.1% incidence. Therefore, these SADRs might not be easily discovered in this study. Hence, an even larger sample size population study would be needed in the future. Second, the medical centers participating in this study were almost first-class hospitals in China which could not represent all-level hospitals, therefore the physicians' skill and patients' bias would exist. Third, since the total ADRs, especially bivalirudin related bleeding events and bivalirudin related thrombocytopenia, rarely occurred, the risk factors analyzed by logistic regression analysis might have relatively low statistical power. Fourth, a long-term follow-up could be conducted to explore the safety of bivalirudin.

## Conclusion

In conclusion, bivalirudin is well-tolerated as anticoagulant for a PCI procedure; meanwhile, older age, renal function impairment, and CRUSADE high risk stratification serve as independent risk factors of bivalirudin related ADRs.

## Data Availability Statement

The original contributions presented in the study are included in the article/Supplementary Material, further inquiries can be directed to the corresponding author.

## Ethics Statement

The studies involving human participants were reviewed and approved by the Ethics Committee of Beijing Anzhen Hospital Affiliated to Capital Medical University (KS2019012) and Peking University People's Hospital (2018PHA092-001). The patients/participants provided their written informed consent to participate in this study.

## Author Contributions

PL and HZ made substantial contributions to the design of the present study. Data acquisition and interpretation were performed by PL, HZ, CL, ZJ, ZZ, ZL, FW, JL, and LH. PL, HZ, JL, and LH critically revised the manuscript for important intellectual content. All authors approved the final version of the manuscript. All authors agree to be accountable for all aspects of the work in ensuring that questions related to the accuracy or integrity of the work are appropriately investigated and resolved.

## Conflict of Interest

The authors declare that the research was conducted in the absence of any commercial or financial relationships that could be construed as a potential conflict of interest.

## Publisher's Note

All claims expressed in this article are solely those of the authors and do not necessarily represent those of their affiliated organizations, or those of the publisher, the editors and the reviewers. Any product that may be evaluated in this article, or claim that may be made by its manufacturer, is not guaranteed or endorsed by the publisher.
