# Supplementary material for: Occurrence and Risk Factors of Adverse Drug Reactions in Patients Receiving Bivalirudin as Anticoagulant During Percutaneous Coronary Intervention: A Prospective, Multi-Center, Intensive Monitoring Study
Source: Front Cardiovasc Med. 2022 Apr 29;8:781632. doi: 10.3389/fcvm.2021.781632 (PMC9099409; doi:10.3389/fcvm.2021.781632)
Supplement: Supplementary file 1 [file Table_1.docx]

**Supplementary Table 1.** Participants in each medical center

| Items | Participants, No. (%) | Full analysis set, No. (%) | Completed the study, No. (%) | Withdrew from the study, No. (%) |
| --- | --- | --- | --- | --- |
| Total | 3050 (100.00) | 3049 (99.97) | 3015 (98.85) | 35 (1.15) |
| Liaoning Provincial People's Hospital | 207 (6.79) | 207 (6.79) | 207 (6.79) | 0 (0.00) |
| Taian Central Hospital of Shandong Province | 321 (10.52) | 321 (10.52) | 320 (10.49) | 1 (0.03) |
| Yulin First people's Hospital | 352 (11.54) | 352 (11.54) | 349 (11.44) | 3 (0.10) |
| Cardiology Department of Tangshan workers' Hospital | 178 (5.84) | 178 (5.84) | 178 (5.84) | 0 (0.00) |
| The First People's Hospital of Jinzhou District, Dalian | 11 (0.36) | 11 (0.36) | 11 (0.36) | 0 (0.00) |
| The First Hospital of Zhangjiakou | 99 (3.25) | 99 (3.25) | 99 (3.25) | 0 (0.00) |
| The First Affiliated Hospital of Nanchang University | 139 (4.56) | 139 (4.56) | 139 (4.56) | 0 (0.00) |
| Peking University People's Hospital | 36 (1.18) | 36 (1.18) | 36 (1.18) | 0 (0.00) |
| Jinan Central Hospital | 63 (2.07) | 63 (2.07) | 57 (1.87) | 6 (0.20) |
| The First Affiliated Hospital of Henan University | 39 (1.28) | 39 (1.28) | 36 (1.18) | 3 (0.10) |
| Chongqing Emergency center | 91 (2.98) | 91 (2.98) | 90 (2.95) | 1 (0.03) |
| Jiaozuo People's Hospital | 81 (2.66) | 81 (2.66) | 80 (2.62) | 1 (0.03) |
| Puyang Oilfield General Hospital | 55 (1.80) | 55 (1.80) | 51 (1.67) | 4 (0.13) |
| Nanchong Central Hospital | 58 (1.90) | 58 (1.90) | 58 (1.90) | 0 (0.00) |
| Jiangxi Provincial People's Hospital | 297 (9.74) | 297 (9.74) | 297 (9.74) | 0 (0.00) |
| Tianjin Chest Hospital | 20 (0.66) | 20 (0.66) | 20 (0.66) | 0 (0.00) |
| Wuzhou People's Hospital | 51 (1.67) | 51 (1.67) | 51 (1.67) | 0 (0.00) |
| The Second Affiliated Hospital of Nanchang University | 90 (2.95) | 90 (2.95) | 90 (2.95) | 0 (0.00) |
| Lvcheng Cardiovascular Hospital | 39 (1.28) | 39 (1.28) | 39 (1.28) | 0 (0.00) |
| Mianyang Central Hospital | 183 (6.00) | 183 (6.00) | 182 (5.97) | 1 (0.03) |
| Xuchang Central Hospital | 99 (3.25) | 99 (3.25) | 89 (2.92) | 10 (0.33) |
| Chenzhou First People's Hospital | 63 (2.07) | 62 (2.03) | 62 (2.03) | 1 (0.03) |
| Xintai People's Hospital | 237 (7.77) | 237 (7.77) | 237 (7.77) | 0 (0.00) |
| Taian First People's Hospital | 58 (1.90) | 58 (1.90) | 58 (1.90) | 0 (0.00) |
| Zhoukou Central Hospital | 41 (1.34) | 41 (1.34) | 39 (1.28) | 2 (0.07) |
| Xuzhou Central Hospital | 115 (3.77) | 115 (3.77) | 114 (3.74) | 1 (0.03) |
| Beijing Anzhen Hospital Affiliated to Capital Medical University | 27 (0.89) | 27 (0.89) | 26 (0.85) | 1 (0.03) |
