# Supplementary material for: Occurrence and Risk Factors of Adverse Drug Reactions in Patients Receiving Bivalirudin as Anticoagulant During Percutaneous Coronary Intervention: A Prospective, Multi-Center, Intensive Monitoring Study
Source: Front Cardiovasc Med. 2022 Apr 29;8:781632. doi: 10.3389/fcvm.2021.781632 (PMC9099409; doi:10.3389/fcvm.2021.781632)
Supplement: Supplementary file 2 [file Table_2.docx]

**Supplementary Table 2.** Characteristics of PCI

| Parameters | Patients |
| --- | --- |
| Operative timing, No. (%) |  |
| Emergency operation | 1351 (44.31) |
| Elective operation | 1698 (55.69) |
| Types of coronary interventional therapy, No. (%) |  |
| Stent implantation | 2915 (95.60) |
| Balloon dilatation | 121 (3.97) |
| Thrombus aspiration | 0 (0.00) |
| Others | 13 (0.43) |
| Arterial access, No. (%) |  |
| Radial artery | 2884 (94.59) |
| Femoral artery | 155 (5.08) |
| Brachial artery | 2 (0.07) |
| Others | 8 (0.26) |
| Operative duration (minutes), mean±SD | 69.60±48.00 |
| Duration of bivalirudin infusion (minutes), mean±SD | 91.15±77.40 |
| Full-dose use of bivalirudin, No. (%) |  |
| Yes | 1942 (63.69) |
| No | 1107 (36.31) |
| Glycoprotein IIb/IIIa inhibitor use, No. (%) |  |
| Yes | 2221 (72.84) |
| No | 828 (27.16) |
| TIMI flow grade, No. (%) |  |
| Pre-procedure |  |
| 0 | 939 (30.80) |
| 1 | 501 (16.43) |
| 2 | 285 (9.35) |
| 3 | 1316 (43.16) |
| Unknown | 8 (0.26) |
| Post-procedure |  |
| 0 | 7 (0.23) |
| 1 | 14 (0.46) |
| 2 | 41 (1.34) |
| 3 | 2985 (97.90) |
| Unknown | 2 (0.07) |

PCI, percutaneous coronary intervention; TIMI, Thrombolysis in Myocardial Infarction; SD, standard deviation.
