# Supplementary material for: Occurrence and Risk Factors of Adverse Drug Reactions in Patients Receiving Bivalirudin as Anticoagulant During Percutaneous Coronary Intervention: A Prospective, Multi-Center, Intensive Monitoring Study
Source: Front Cardiovasc Med. 2022 Apr 29;8:781632. doi: 10.3389/fcvm.2021.781632 (PMC9099409; doi:10.3389/fcvm.2021.781632)
Supplement: Supplementary file 3 [file Table_3.docx]

**Supplementary Table 3.** Clinical administration of bivalirudin

| Items | Before operation | During operation | Within 4 h after operation | More than 4 h after operation |
| --- | --- | --- | --- | --- |
| Dosages of bivalirudin |  |  |  |  |
| 0.75 mg/kg | 2030 (66.58) | 579 (18.99) | 2 (0.07) | 0 (0.00) |
| 0.3 mg/kg | 0 (0.00) | 0 (0.00) | 0 (0.00) | 0 (0.00) |
| 1.75 mg/kg/h | 4 (0.13) | 2651 (86.95) | 2372 (77.80) | 41 (1.34) |
| 1.0 mg/kg/h | 0 (0.00) | 3 (0.10) | 134 (4.39) | 1 (0.03) |
| 0.2 mg/kg/h | 0 (0.00) | 0 (0.00) | 102 (3.35) | 199 (6.53) |
| Other dosage | 382 (12.53) | 394 (12.92) | 339 (11.12) | 1 (0.03) |
| Method of administration |  |  |  |  |
| Intravenous injection | 2412 (79.11) | 578 (18.96) | 1 (0.03) | 0 (0.00) |
| Intravenous drip | 4 (0.13) | 3046 (99.90) | 2938 (96.36) | 242 (7.94) |
| Interrupt administration |  |  |  |  |
| Yes | 0 (0.00) | 10 (0.33) | 2 (0.07) | 0 (0.00) |
| No | 2412 (79.11) | 3039 (99.67) | 2936 (96.29) | 242 (7.94) |
