# Supplementary material for: Occurrence and Risk Factors of Adverse Drug Reactions in Patients Receiving Bivalirudin as Anticoagulant During Percutaneous Coronary Intervention: A Prospective, Multi-Center, Intensive Monitoring Study
Source: Front Cardiovasc Med. 2022 Apr 29;8:781632. doi: 10.3389/fcvm.2021.781632 (PMC9099409; doi:10.3389/fcvm.2021.781632)
Supplement: Supplementary file 4 [file Table_4.docx]

**Supplementary Table 4.** Detailed AEs in System Organ Class (SOC)

| Items | Total | | SAEs | | Death due to AEs | |
| --- | --- | --- | --- | --- | --- | --- |
|  | Number of times | Incidence, No. (%) | Number of times | Incidence, No. (%) | Number of times | Incidence, No. (%) |
| Total | 829 | 414 (13.58) | 38 | 31 (1.02) | 9 | 8 (0.26) |
| Gastrointestinal disorders | 158 | 109 (3.58) | 9 | 8 (0.26) | 0 | 0 (0.00) |
| General disorders and administration site conditions | 123 | 101 (3.31) | 5 | 5 (0.16) | 2 | 2 (0.07) |
| Respiratory, thoracic, and mediastinal disorders | 138 | 97 (3.18) | 2 | 2 (0.07) | 1 | 1 (0.03) |
| Blood and lymphatic system disorders | 81 | 81 (2.66) | 1 | 1 (0.03) | 0 | 0 (0.00) |
| Investigations | 68 | 54 (1.77) | 2 | 2 (0.07) | 1 | 1 (0.03) |
| Cardiac disorders | 61 | 48 (1.57) | 12 | 12 (0.39) | 5 | 5 (0.16) |
| Nervous system disorders | 47 | 40 (1.31) | 4 | 4 (0.13) | 0 | 0 (0.00) |
| Renal and urinary disorders | 35 | 30 (0.98) | 0 | 0 (0.00) | 0 | 0 (0.00) |
| Skin and subcutaneous tissue disorders | 22 | 21 (0.69) | 0 | 0 (0.00) | 0 | 0 (0.00) |
| Infections and infestations | 17 | 16 (0.52) | 1 | 1 (0.03) | 0 | 0 (0.00) |
| Musculoskeletal and connective tissue disorders | 18 | 14 (0.46) | 1 | 1 (0.03) | 0 | 0 (0.00) |
| Metabolism and nutrition disorders | 14 | 13 (0.43) | 1 | 1 (0.03) | 0 | 0 (0.00) |
| Hepatobiliary disorders | 13 | 11 (0.36) | 0 | 0 (0.00) | 0 | 0 (0.00) |
| Psychiatric disorders | 11 | 10 (0.33) | 0 | 0 (0.00) | 0 | 0 (0.00) |
| Vascular disorders | 11 | 10 (0.33) | 0 | 0 (0.00) | 0 | 0 (0.00) |
| Eye disorders | 4 | 4 (0.13) | 0 | 0 (0.00) | 0 | 0 (0.00) |
| Reproductive system and breast disorders | 2 | 2 (0.07) | 0 | 0 (0.00) | 0 | 0 (0.00) |
| Injury, poisoning and procedural complications | 3 | 2 (0.07) | 0 | 0 (0.00) | 0 | 0 (0.00) |
| Endocrine disorders | 2 | 2 (0.07) | 0 | 0 (0.00) | 0 | 0 (0.00) |
| Immune system disorders | 1 | 1 (0.03) | 0 | 0 (0.00) | 0 | 0 (0.00) |

AEs, adverse events; SAEs, severe adverse events.
