# Supplementary material for: Occurrence and Risk Factors of Adverse Drug Reactions in Patients Receiving Bivalirudin as Anticoagulant During Percutaneous Coronary Intervention: A Prospective, Multi-Center, Intensive Monitoring Study
Source: Front Cardiovasc Med. 2022 Apr 29;8:781632. doi: 10.3389/fcvm.2021.781632 (PMC9099409; doi:10.3389/fcvm.2021.781632)
Supplement: Supplementary file 5 [file Table_5.docx]

**Supplementary Table 5.** Summary of bleeding events in System Organ Class (SOC)

| Items | Total bleeding events | | Bleeding events related to study drug | |
| --- | --- | --- | --- | --- |
|  | Number of times | Incidence, No. (%) | Number of times | Incidence, No. (%) |
| Total | 147 | 126 (4.13) | 37 | 34 (1.12) |
| Gastrointestinal disorders | 75 | 68 (2.23) | 23 | 21 (0.69) |
| Respiratory, thoracic, and mediastinal disorders | 27 | 27 (0.89) | 5 | 5 (0.16) |
| Renal and urinary disorders | 12 | 11 (0.36) | 4 | 4 (0.13) |
| General disorders and administration site conditions | 10 | 10 (0.33) | 1 | 1 (0.03) |
| Investigations | 6 | 6 (0.20) | 2 | 2 (0.07) |
| Skin and subcutaneous tissue disorders | 4 | 4 (0.13) | 0 | 0 (0.00) |
| Infections and infestations | 3 | 3 (0.10) | 0 | 0 (0.00) |
| Vascular disorders | 3 | 2 (0.07) | 0 | 0 (0.00) |
| Eye disorders | 2 | 2 (0.07) | 0 | 0 (0.00) |
| Blood and lymphatic system disorders | 2 | 2 (0.07) | 1 | 1 (0.03) |
| Cardiac disorders | 1 | 1 (0.03) | 0 | 0 (0.00) |
| Nervous system disorders | 1 | 1 (0.03) | 0 | 0 (0.00) |
| Injury, poisoning and procedural complications | 1 | 1 (0.03) | 1 | 1 (0.03) |
