# Supplementary material for: Occurrence and Risk Factors of Adverse Drug Reactions in Patients Receiving Bivalirudin as Anticoagulant During Percutaneous Coronary Intervention: A Prospective, Multi-Center, Intensive Monitoring Study
Source: Front Cardiovasc Med. 2022 Apr 29;8:781632. doi: 10.3389/fcvm.2021.781632 (PMC9099409; doi:10.3389/fcvm.2021.781632)
Supplement: Supplementary file 6 [file Table_6.docx]

**Supplementary Table 6.** New ADRs in System Organ Class (SOC)

| Items | Number of times | Incidence, No. (%) |
| --- | --- | --- |
| Total | 8 | 7 (0.23) |
| Skin and subcutaneous tissue disorders | 2 | 2 (0.07) |
| Gastrointestinal disorders | 1 | 1 (0.03) |
| Investigations | 1 | 1 (0.03) |
| Respiratory, thoracic, and mediastinal disorders | 1 | 1 (0.03) |
| Musculoskeletal and connective tissue disorders | 1 | 1 (0.03) |
| Cardiac disorders | 1 | 1 (0.03) |
| Nervous system disorders | 1 | 1 (0.03) |

ADRs, adverse drug reactions.
